# Effectiveness of a 6-week specific rehabilitation program combining education and exercises on walking capacity in patients with lumbar spinal stenosis with neurogenic claudication: a randomized controlled clinical trial protocol

**DOI:** 10.1186/s13063-022-07011-3

**Published:** 2022-12-27

**Authors:** Mariève Houle, Charles Tétreau, Claude-Édouard Châtillon, Andrée-Anne Marchand, Martin Descarreaux

**Affiliations:** 1 Department of Anatomy, Université du Québec à Trois-Rivières, 3351, boul. des Forges, Trois-Rivières, QC G8Z 4M3 Canada; 2 Department of Human Kinetics, Université du Québec à Trois-Rivières, Trois-Rivières, Canada; 3 Centre intégré universitaire de santé et de services sociaux de la Mauricie et du Centre-du-Québec (CIUSSS MCQ), Trois-Rivières, Canada; 4 Division of Neurosurgery, Faculty of Medicine, University of Montreal, Montréal, Canada; 5 Department of Chiropractic, Université du Québec à Trois-Rivières, Trois-Rivières, Canada

**Keywords:** Neurogenic claudication, Walking capacity, Exercises, Education, Spinal stenosis, Randomized controlled trial

## Abstract

**Background:**

Lumbar spinal stenosis (LSS) causing neurogenic claudication (NC) is a leading cause of disability which is intimately related to a decrease in walking capacity. Walking limitation has negative physical and mental impacts on patients. Recent guidelines recommend the use of conservative treatment options such as exercises before considering surgery. Unfortunately, dedicated healthcare resources for the conservative management of patients with LSS causing NC are uncommon. Thus, it is important to develop accessible and specific rehabilitation programs aimed at improving patients’ self-management, especially with regard to walking capacity. The aim of this study is to evaluate the effectiveness of a 6-week specific rehabilitation program combining education and exercises on walking capacity in patients with LSS causing NC.

**Methods/design:**

This is a prospective randomized controlled parallel-group clinical trial. Sixty-six patients with LSS causing NC will be recruited from identified clinics and local advertisements. The intervention group will receive standardized education and specific exercises while the control group will only receive a standardized education. The program in both groups will last for 6 weeks with 5 evaluation time points (baseline, week 2, week 4, week 6, and week 12). The primary outcome will be walking capacity measured with the Self-Paced Walking Test, and the secondary outcomes will be back and leg pain intensity, LSS-related disability, self-efficacy, level of physical activity, anxiety, depression, gait pattern characteristics, balance, and global perceived change.

**Discussion:**

This study will assess the effectiveness of a 6-week specific rehabilitation program combining education and exercises on walking capacity in patients with LSS causing NC. By measuring objective gait pattern characteristics, the study will also provide new information about the impact of NC on gait pattern that could eventually improve the evaluation and the management of LSS.

**Trial registration:**

ClinicalTrials.gov NCT05513326. Registered on August 22, 2022

**Supplementary Information:**

The online version contains supplementary material available at 10.1186/s13063-022-07011-3.

## Background

Neurogenic claudication (NC) is a clinical syndrome recognized as the hallmark of symptomatic lumbar spinal stenosis (LSS) [1], a degenerative musculoskeletal condition caused by age-related changes in the lower spine and defined as the narrowing of the spinal canal or intervertebral foramina [2, 3]. Neurogenic claudication is characterized by bilateral or unilateral buttock, thigh or leg pain, and discomfort such as numbness, tingling, and weakness [4–7]. These symptoms are exacerbated by prolonged standing or walking, and they are temporarily relieved by sitting and bending forward [1, 6]. Because of its symptomatology, especially during daily activities that require walking, NC represents one of the leading causes of pain and disability in the elderly [8].

Over the past years, decreased walking capacity in patients with LSS causing NC, measured in time or distance, has been well documented using objective walking tests (treadmill and self-paced walking tests) [9, 10]. Moreover, a recent systematic scoping review reported that walking capacity was negatively associated with pain outcomes and disability [11]. In addition, greater disability scores were negatively associated with walking speed and stride length, two important gait pattern characteristics of LSS [11]. Patients consider walking capacity and pain to be the most bothersome consequences of NC. They also report that decreased walking capacity is responsible for disrupting their daily life activities (e.g., walking, social activities, household activities, sleeping, and lifting) [12]. Furthermore, the decline in walking capacity due to NC is reflected in an increase in inactivity. Adoption of such a sedentary lifestyle contributes to a decline in quality of life especially with respect to physical status [13, 14]. Previous studies showed that patients with LSS causing NC are less active than the general population over the age of 60 and individuals with hip or knee osteoarthritis, with approximately 82% of their time spent in sedentary behavior [13–15].

Walking limitations related to LSS causing NC do not only affect patients physically. When asked about their well-being and psychological state, most patients report that they feel sad, discouraged, and/or anxious and that they lost interest in doing certain activities because of their condition [12]. However, there are many different ways to assess psychological factors including anxiety and depression in both clinical and research settings. Due to the lack of standardization regarding the assessment of psychological factors, the association between walking capacity and psychological factors in patients with LSS causing NC remains uncertain [11].

Walking limitations and pain related to NC are central to the management of LSS [1, 12, 16]. Several therapeutic options are available for patients with LSS causing NC including conservative approaches and surgical options. According to recent practice guidelines, surgical options should only be considered when less-invasive treatments such as manual therapy, physiotherapy, exercises, and injections have failed to improve symptoms [17]. Having additional resources such as exercises and education in patients’ healthcare trajectories could help them to at least maintain their functional capacities. Furthermore, underlying medical conditions may limit surgical options in this population. To date, dedicated programs providing active treatments such as exercises to patients with LSS causing NC are scarce. Furthermore, the often protracted delay between diagnosis and eventual surgery may be an opportunity to help patients improve both their pre- and post-surgical functional capacities.

Neurogenic claudication related to LSS can be experienced differently over time and between patients, especially regarding leg pain intensity, time to first symptoms when walking, and total walking time. Knowing that there are currently no specific resources to guide active treatment such as exercises for patients with LSS, identifying and developing specific and flexible rehabilitation programs to help patients to maintain or improve their walking capacity would address an important knowledge gap. Such a program would offer an additional access to healthcare during patients’ care trajectories in addition to offering a chance for patients to self-manage their condition over time. This new opportunity should help them strengthen their self-efficacy during daily living.

Bove and colleagues reported that patients value individual sessions focused on their specific impairments and limitations as well as specific tips on self-management strategies [18]. In the last few years, RCTs involving patients with LSS causing NC were designed to include education as part of the intervention [19–22]. However, the authors did not describe in detail the content of the education program (i.e., daily living tips to limit pain, information regarding walking and sleeping) nor how it was delivered during the study (e.g., frequency, duration, delivery methods such as individual session or information flyers) making the replication of those studies difficult.

The aim of the proposed protocol is to evaluate the effectiveness of a 6-week specific rehabilitation program combining education and exercises on walking capacity in patients with LSS causing NC. It is hypothesized that a 6-week specific rehabilitation program combining exercises and education will have a superior effect on walking capacity than education alone.

## Methods/design

### Study design

**Fig. 1:**
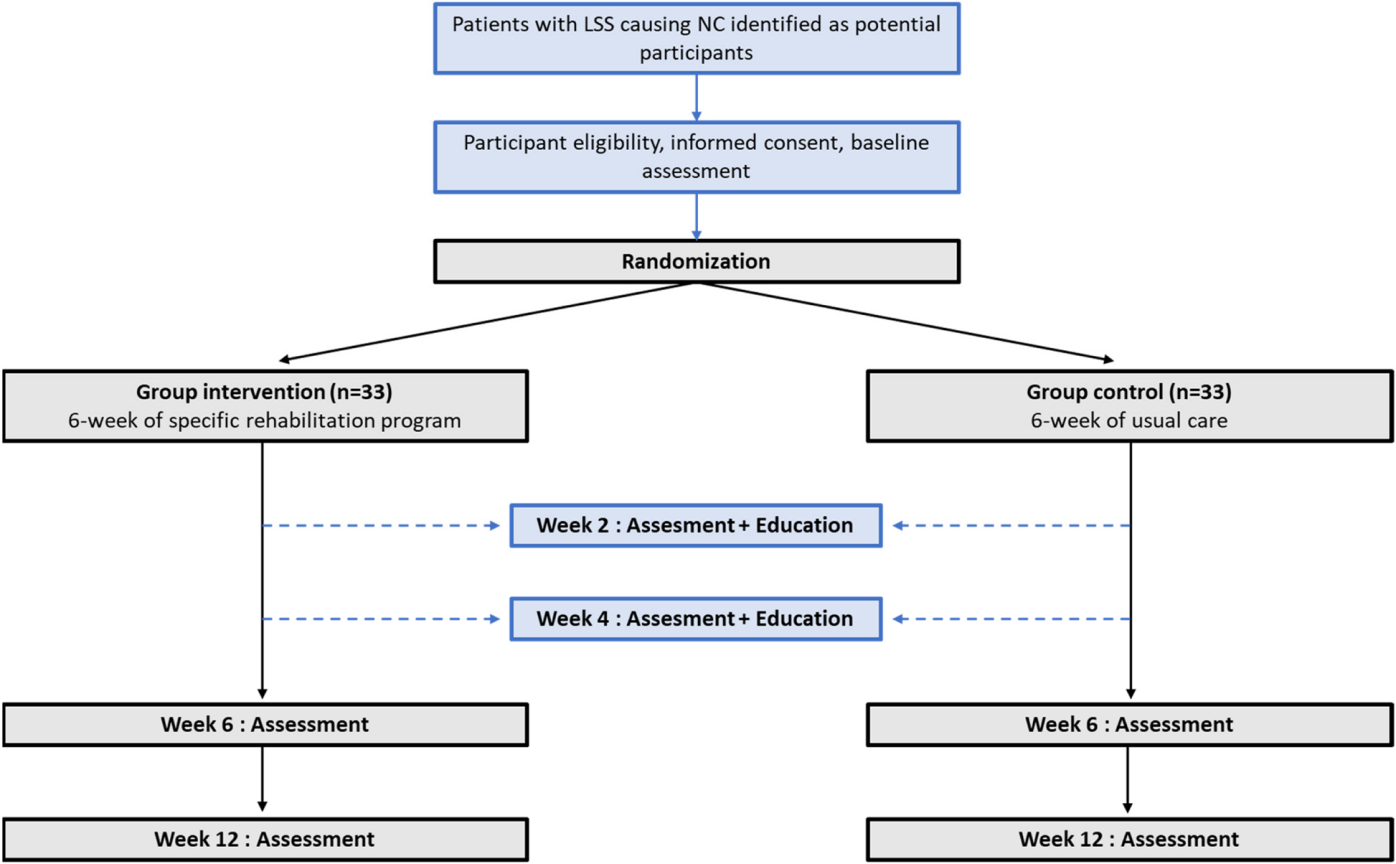
Study timeline. LSS = lumbar spinal stenosis, NC = neurogenic claudication

The proposed study is a prospective randomized controlled parallel-group clinical trial (RCT) that aims to evaluate the superior effect of a 6-week specific rehabilitation program combining exercises and education compared to education alone (see Fig. 1). In this RCT, participants with LSS causing NC will be allocated to either the intervention group (LSS group) or the control group. All proposed methods are in accordance with the CONSORT statement which provides relevant guidelines and regulations for RCTs [23]. The study protocol is written in conformity with the Standard Protocol Items: Recommendations for Interventional Trials (SPIRIT) statement [24] and has been registered on ClinicalTrials.gov (NCT05513326) on August 22, 2022.

### Recruitment

Potential participants will be identified by neurosurgeons and chiropractors from collaborating clinics during regular scheduled appointments or by advertising in the local newspapers or via social media. Both recruitment strategies (pamphlets given by clinicians or local publicity) will include information regarding the study objectives, patient implication, and contact information of the trial coordinator. If interested, participants will be screened for eligibility criteria over the phone, and then an assessment date will be set. Collaborating clinics are located in Trois-Rivières (Québec, Canada) and refer to the chiropractic university teaching clinic and to the regional hospital neurosurgery outpatient clinic.

#### Eligibility criteria

To be eligible, participants must meet the following inclusion criteria: (1) being at least 50 years old; (2) having a degenerative central LSS alone or in combination with other LSS types (e.g., foraminal, lateral recess) and/or a spondylolisthesis affecting one or multiple vertebral levels, confirmed by clinical history, physical examination, and proper imaging (MRI and/or CT scan); (3) having NC associated with LSS; (4) with a duration of signs and symptoms of at least 3 months; (5) being able to walk at least 20 m without walking aid, but not being able to walk continuously for 30 min; (6) being willing to attend 3 intervention sessions per week over a period of 6 weeks; (7) being able to speak and understand French; and (8) being able to provide informed written consent. Participants with congenital LSS, traumatic and pathological causes of LSS, symptomatic osteoarthritis (hip or knee) causing limited walking capacity, a neurological disease affecting walking capacity such as Parkinson, uncontrolled diabetes, heart failure, intermittent claudication of vascular origin, impaired cognitive capacity, and history of back or lower extremities surgery in the past 3 months will be excluded.

### Ethical considerations

This study will be conducted according to the Helsinki Declaration. The proposed study protocol received approval from the CIUSSS MCQ research ethics committee (CÉR-2023-649) and from the Human Research Ethics Board of UQTR which recognizes the ethics approval first received from the CIUSSS MCQ. Participants will be informed that they can withdraw at any moment during the study without having to provide an explanation and without negative consequences on their healthcare trajectory. Participants will be asked for permission from the research team to share relevant data with people from the universities taking part in the research or from regulatory authorities, when relevant. Also, it is important to note that if a participant is finally considered by a neurosurgeon for surgery, the proposed protocol will not interfere or delay the surgery. Participants randomized to the control group (education alone) will be offered the specific rehabilitation program after the completion of the study. Written informed consent will be obtained by all participants before completing the baseline assessment. This study does not involve the collection of biological specimens for storage.

### Randomization

All included participants will be randomized into one of the two groups (intervention or control) using a minimization method (allocation ratio of 1:1) to ensure that both groups will be balanced with respect to predetermined criteria. These minimization criteria have been identified based on factors known to have a negative influence on walking capacity and include smoking, diabetes, chronic obstructive pulmonary disease, and stroke. All criteria will have the same weight in the minimization process. Participants will be randomized using a computer-generated list of random numbers which will be placed into sealed and opaque envelopes numbered by an independent research assistant that is not involved in the selection, intervention, or assessment of participants. Each envelope will be opened in front of the participants at the end of the baseline assessment by another independent research assistant.

### Blinding

The researchers performing data collection as well as those performing statistical analyses will be blinded to the group allocation.

### Interventions

#### Intervention group

Participants allocated to the intervention group will receive a 6-week specific rehabilitation program which includes training and education sessions. The one-on-one training sessions will be composed of specific exercises to be performed 3 times per week under the supervision of a certified kinesiologist. Of the three training sessions, one will take place at UQTR’s outpatient clinic while the other two will take place online using a telecommunication platform (e.g., Zoom or Google Duo, depending on the participant’s preference) allowing for remote supervision of the training by the certified kinesiologist. Instructions regarding the selected telecommunication platform will be provided to the participants. Education will take place at week 2 and week 4 with the researcher responsible of the assessments. The presence to exercises and education sessions will be monitored by the responsible research team member (CT for exercises and MH for education).

##### Exercises

Each training session will last 30 min with a focus on lower limb muscle strengthening as well as balance. Sessions will start with 5 min of activation (walking on a treadmill or cycling). Then, participants will be asked to complete 5 exercises targeting lower limb strengthening (time: 18 min) followed by 2 exercises designed to improve balance and 3 stretching exercises for the lower limb (time: 7 min). Levels of difficulty for each exercise will vary according to the patient’s capacity to ensure that each exercise is appropriate and safe while allowing a progression over time.

##### Education

Education will consist of two one-on-one sessions (during week 2 and week 4 assessments) during which participants will receive information directly related to LSS and NC using pre-recorded standardized videos developed for the study. This information consists of explanations of the pathophysiology of LSS and NC as well as expected natural history, usual symptoms, pain management strategies for daily living (body positioning when walking and when sleeping), and physical activity recommendations. Illustrations and videos will be used to enhance participants’ understanding and to ensure a standardized delivery of education. Participants will also be invited to ask questions. Each session will last for 20 min.

#### Control croup

The participants in the control group will only receive the education sessions at week 2 and at week 4.

##### Participant trajectories

Regardless of group allocation, all participants will continue to follow their usual care routine. Usual care can include general advice for daily living; advice to stay active; oral medication to control pain, inflammation, or mood; and a referral for a spinal injection. Their treatments will be monitored throughout the study duration.

### Data collection

Participants will be evaluated five times during the study (baseline, week 2, week 4, week 6, and week 12). Each assessment will include both patient-reported outcome measures (PROMs) and objective physical measures. All assessments will be conducted at the UQTR biomechanics laboratory. The data collection process is synthesized in Table 1. All participants will be called for a reminder before each assessment.

**Table 1:** Data collection

	Measures	Baseline assessment	Week 2 assessment	Week 4 assessment	Week 6 assessment	Week 12 assessment
Baseline measurements	Sociodemographic data and physical examination	X	–	–	–	–
Primary outcome	SPWT	X	X	X	X	X
Secondary outcomes	NRS for leg pain intensity	X	X	X	X	X
	NRS for back pain intensity	X	X	X	X	X
	Leg pain dominance	X	–	–	–	–
	FC-SSSQ	X	–	–	X	X
	FC-CPSES	X	–	–	X	X
	FC-HADS	X	–	–	X	X
	QAPPA	X	–	–	X	X
	Gait pattern characteristics	X	X	X	X	X
	SPPB	X	–	–	X	X
	Global perceived change	–	X	X	X	X

*SPWT* Self-Paced Walking Test, *NRS* Numerical Rating Scale, *FC-SSSQ* French-Canadian adaptation of the Swiss Spinal Stenosis Questionnaire, *FC-CPSES* French-Canadian Chronic Pain Self-Efficacy Scale, *FC-HADS* French-Canadian adaptation of the Hospital Anxiety and Depression Scale, *QAPPA* Questionnaire d’activité physique pour les personnes âgées [Physical Activity Questionnaire for the Elderly], *SPPB* Short Physical Performance Battery

During the baseline assessment, participants will first complete a series of questionnaires and undergo a standardized physical examination. The questionnaires will include measures of age, sex, height, weight, leg pain dominance, and number of falls in the past 6 months. Participants will also be asked about the presence of comorbidities known to have a negative influence on walking capacity as well as previous conservative treatment (e.g., injection, manual therapy). The physical examination will include the assessment of lower limb dermatomes and myotomes as well as pallesthesia and osteotendinous reflexes (to document the absence of peripheral neuropathy).

### Primary outcome

The primary outcome of this study is walking capacity which will be assessed using the Self-Paced Walking Test (SPWT). The SPWT represents the current gold standard for assessing walking capacity in patients with LSS [9, 25]. During this test, participants will be asked to walk freely on a leveled surface at their own pace until they are forced to stop for a minimum of 3 s because of LSS symptoms. The time and distance reached by the participants will be measured. The minimal clinically important change (MCID) reported for the SPWT varies between 319 and 376 m [26]. As patients with LSS causing NC are at higher risk of falls, participants will walk near a ramp and a research assistant will also walk behind the participant during the walking test to assist if needed.

### Secondary outcomes

#### Patients-reported outcomes measures

Participants will be invited to complete a set of questionnaires assessing physical and psychological PROMs including leg and back pain (numerical rating scale) [27], LSS-related disability (French-Canadian version of the Swiss Spinal Stenosis) [28], self-efficacy (French-Canadian Chronic Pain Self-Efficacy Scale) [29], physical activity (Questionnaire d’activité physique pour les personnes âgées) [30], and anxiety and depression (French-Canadian adaptation of the Hospital Anxiety and Depression Scale) [31].

##### Leg and back pain

Leg and back pain intensity will be independently assessed using an 11-point Numerical Rating Scale (NRS) (0 indicating an absence of pain, 10 indicating the worst pain imaginable) [27].

##### Disability-related to LSS

LSS-related disability will be measured using an adapted version of the validated French-Canadian version of the Swiss Spinal Stenosis Questionnaire (FC-SSSQ) [28]. This questionnaire is a LSS-specific tool composed of three subscales which assess symptom severity, physical function, and satisfaction with care. The pain subscale includes seven items of which six are scored using a 5-point Likert scale, and one (Q7) is scored using 1, 3, or 5 points. The physical function subscale includes 5 items scored on a 4-point Likert scale. In the proposed study, the satisfaction subscale will be removed because no participants will have undergone surgery. For the symptom severity and physical function subscales, a higher score indicates greater disability, and the FC-SSSQ total possible score without considering the satisfaction subscale is 55 points. We considered that this outcome measure is the most appropriate for patients with LSS causing NC. In fact, the SSSQ has been established as the gold standard questionnaire to assess pain and function [32]. This questionnaire is also highly correlated with the SPWT [33], the standard criterion to assess walking capacity in patients with LSS causing NC [9].

##### Self-efficacy

Self-efficacy will be assessed using the validated French-Canadian Chronic Pain Self-Efficacy Scale (FC-CPSES) [29]. Each of its 33 items is measured using a 10-point numerical scale (1 = not at all confident and 10 = totally confident). The FC-CPSES total score ranges from 1 to 10 and is obtained by calculating the mean score of the 33 items. Higher scores indicate higher self-efficacy.

##### Physical activity

Physical activity level will be assessed using the validated *Questionnaire d’activité physique pour les personnes âgées* [Physical Activity Questionnaire for the Elderly] (QAPPA). This questionnaire assesses the amount of moderate and vigorous physical activity during the last 7 days (MET-min/week). Following the completion of this questionnaire, participants can be classified into the following categories according to their physical activity volume: high, moderate, or low physical activity level [30, 34].

##### Anxiety and depression

Anxiety and depression will be assessed using the validated French-Canadian adaptation of the Hospital Anxiety and Depression Scale (FC-HADS) [31]. This questionnaire is a 14-item scale with 7 items assessing anxiety and 7 items assessing depression. Each item is scored from 0 to 3 for a maximum of 21 points for each subscale. A higher score indicates higher anxiety (HADS-A) or higher depression (HADS-D).

##### Patients’ global impression of change

Patient’s global impression of change (PGIC) will be measured using a 7-point scale that ranges from “very much improved” to “very much worse” with “no change” as the mid-point [35, 36].

#### Objectives measures

##### Walking capacity and gait pattern characteristics

During the SPWT, two synchronized inertial sensors (Physilog®6 – GaitUp, Lausanne, Switzerland, 128 Hz) will be placed and attached on the top of each participant’s shoe. These inertial sensors include a 3-dimension accelerometer, gyroscope, and magnetometer which allow the measurement of more than 25 gait parameters including general (e.g., speed, left/right asymmetry, variability, and cycle duration), temporal (e.g., stance, swing, and double support), spatial (e.g., swing speed, swing width, and strike angle), and clearance (maximum heel, maximum and minimum toe clearance) parameters.

##### Lower extremity physical function and balance

Lower extremity physical function and balance will be assessed using the Short Physical Performance Battery (SPPB), a validated test battery among older adults [37]. This test battery includes standing balance, 4-m walking at the usual pace, and repeated sit-to-stand tests. Each of the three tests of the battery is scored from 0 (inability to perform) to 4 (high category of performance) which puts the total score out of 12 (a higher score indicates lower extremity function). The standing balance test is divided into three components: side-by-side, semi-tandem, and tandem balance. For each component of the standing balance test, participants are asked to maintain the position for a maximum of 10 s. The 4-m walking test at the usual pace consists of walking at the usual pace on a leveled surface 4-m hallway. Participants will be asked to perform it twice, and the result of the fastest trial will be kept for analysis. The repeated sit-to-stand test consists of performing five sit-to-stand repetitions as fast as possible with arms across the chest. The score for this test is based on the time required to perform five repetitions. If participants are unable to complete five repetitions or if it takes more than 1 min to perform them, the score attributed to this test will be 0.

### Sample size

The sample size for this study has been determined based on the continuous primary outcome (walking capacity) using the minimal clinically important change (MCID) of 331 m which represents the MCID at 2 months following nonsurgical treatments according to Carlesso and colleagues [26]. Considering a one-sided significance level of *α* = 0.05, a power of 80%, and an attrition rate of 15%, a total of 66 participants (33 per group) will need to be recruited.

### Statistical analyses

Baseline characteristics and outcome measures will be summarized with descriptive statistics for each group. Two-tailed independent sample *t* tests will be performed to compare group baseline characteristics. Paired factor analyses using analyses of covariance will be performed for each of the outcome measures (primary and secondary). Such analysis ensures that identified confounding factors do not influence intergroup differences. ANOVAs will be used to compare the evolution of walking capacity over weeks. An intention-to-treat method will be used, and missing data will be replaced using a multiple imputation method. No additional analyses (e.g., subgroup and adjusted analyses) are planned for this study. Statistical analyses will be conducted using SPSS statistical version 26 for the Windows software, and the level of significance will be set at a *p*-value ≤ 0.05.

### Adverse events and harms

All adverse events (minor or major) associated with both intervention and usual care will be monitored using open-ended questions. Adverse events are defined as any undesirable experience occurring to a participant, with or without relation to the intervention. These undesirable experiences include an increase in back or leg pain intensity and claudication discomfort. Each adverse event reported by participants throughout the study will be considered and discussed with the research team. Adverse events that are associated with an increase in disability will be reported to the ethics committees. There is no anticipated harm and compensation for trial participation.

### Data management

The researchers shall fill in data to the data collection sheet accurately, completely, and timely based on original observations. MH will be responsible to fill in data collection sheets during each evaluation, and CT will be responsible to fill in data collection sheets during each intervention. Researchers are responsible for ensuring the accuracy of all data entered and recorded. All data collected during this study are totally confidential and will never allow participant identification. Confidentiality will be assured by replacing the participants’ names by an alphanumeric code. The data collected will be stored under lock and key in the evaluation or intervention room (paper documents) or on a secure network with both network and electronic document access protected by passwords under the responsibility of the Chaire de recherche internationale en santé neuromusculosquelettique at the Université du Québec à Trois-Rivières. Only the research members will have access to these data. All of them have signed a confidentiality agreement. All data will be destroyed 5 years after the end of the study. For monitoring and control purposes, research files may be consulted by a person mandated by the CIUSSS MCQ Research Ethics Board or by a person mandated by authorized public agencies. All these persons and organizations adhere to a confidentiality policy. Full name, contact information, and the start and end date of participation for each participant in the project will be kept for 1 year after the end of the project in a separate directory maintained by the researcher responsible (MH) for this research project. For randomization purposes, a research assistant will have access to baseline data. This research assistant has also signed a confidentiality agreement.

### Plans to give access to the full protocol, participant-level data

Detailed information on the study including study design, eligibility criteria, and outcome measures is currently available to the public on clinical trials. Datasets analyzed during the study, statistical code, and materials are available from the corresponding author upon reasonable request.

### Composition of the coordinating center and trial steering committee and plans for auditing trial conduct

The coordinator of the study is MH. She is responsible for all aspects of the local organization including identifying potential recruits and taking consent from participants. In addition to MH, CEC is responsible for the identification of potential recruits in the regional hospital outpatient clinic. CT (certified kinesiologist) is the supervisor of each session of exercises for the intervention group. The project is supervised by MD and AAM. Both are professors at UQTR and are supervising MH’s doctoral studies. The local Trial Steering Committee is composed of MH, CT, CEC, AAM, and MD. The team discussed the test protocols, the intervention content, and the related materials together. The committee will meet each month throughout the study. If there is any modification to the protocol that needs to be done or if there is any adverse event, the TSC will discuss the situation as soon as possible and will inform the research ethic committee. The monitoring will be conducted by the principal investigator of the study each month as an audit of trial conduct. There is no stakeholder and public involvement group for this study.

### Dissemination plans

The results will be communicated to participants, healthcare professionals, the public, and other relevant groups via publications in scientific journals, platform presentations, and poster presentations during national and international congresses.

## Discussion

The interventions for this RCT were designed according to the best available treatment guidelines and best practice recommendations which include the use of education and an individually tailored supervised exercise program [17, 38]. A recent systematic review focusing on multidisciplinary interventions for chronic pain reported that education should be incorporated into future RCTs because of its contribution to the improvement of chronic pain management especially with regard to self-efficacy. This improvement was observed when education was used as either the active or the control intervention [39]. In addition, a previous qualitative study reported that short bouts of education about exercise techniques improved symptoms related to LSS causing NC [40]. According to Backstrom and colleagues, education should include information related to LSS such as its definition, symptoms manifestations, the natural course of the condition, the purpose of home exercises, information related to pathophysiology, and self-management strategies [41]. Self-management strategies are essential in the management of persistent musculoskeletal conditions such as LSS and should be an important part of education programs [42]. Recently, patient education under many forms (e.g., pathoanatomical descriptions, etiologies, self-management strategies, nutrition modifications, and physical activity) has been included in RCTs for LSS [20, 22, 43, 44]. However, there was heterogeneity regarding education content, intervention duration, and outcome measures among the studies making it difficult to draw conclusions on the best parameters of education to be used. Building on previous findings, the present protocol study will provide a standardized education program for both groups. This should help patients better understand their condition and why exercises can be used to improve walking capacity. In addition, the use of a standardized education program will allow us to isolate the effect of the exercises on the improvement of walking capacity in patients with LSS causing NC.

Regarding rehabilitation programs, the effectiveness of exercises alone in improving objective walking capacity is still not well established [17]. A recent systematic review reported low to moderate-quality evidence for multimodal programs including exercises on the improvement of symptoms and function in patients with LSS causing NC [45]. In addition, another previous RCT showed that the use of supervised exercises provided significantly greater improvement in walking distance, physical function, and symptoms severity compared to self-directed exercises [46]. Similarly, another RCT of Comer et al. showed that a self-directed home exercise program including flexion and aerobic exercises punctually prescribed by physiotherapists did not systematically improve symptom severity and was no more effective than education and advice [22]. However, in most previous RCTs, exercise intensity was not tailored to day-to-day symptom severity. Thus, the present protocol is innovative in two respects: it includes standardized education for all participants, and it takes into consideration the day-to-day evolution of symptom severity to provide a tailored exercise program.

Previously reported rehabilitation programs usually included aerobic and lower limb-strengthening exercises [20, 47–50] but did not incorporate balance-strengthening exercises. However, patients with LSS and NC present an increased risk of falls for a variety of reasons, including deconditioning, lower extremity neurological deficits, polypharmacy, environmental hazards, poor vision, and impairments in gait and activities of daily living [51]. In addition, a previous study by Thornes et al. showed that there was a large inter-individual variation in balance between patients with LSS and NC and that gait stability was negatively associated with disability as measured by the ODI [52]. Based on those findings and knowing that balance limitations have been identified in 45 to 65% of patients with LSS [53, 54], the present protocol will include balance exercises to help decrease the risk of falls in patients with LSS causing NC and to contribute to patient’s self-confidence during walking.

Another point to consider when assessing the effectiveness of any intervention is the primary outcome measure. To date, most studies using exercises as the main intervention for LSS used indirect measures of walking capacity such as functional capacity measured by the SSSQ or the Zurich Claudication Questionnaire (ZCQ), leg and back pain intensity, disability measured by the ODI or the global rating of change scale as their primary outcome [46, 48–50]. Considering that a decrease in walking capacity is considered one of the most bothersome consequences of LSS causing NC [12], direct measures of walking capacity using a self-paced walking test should be used when assessing the effectiveness of interventions on functional capacities.

Overall, having a flexible rehabilitation program combining lower limb strengthening and balance exercises should lead to an improvement in walking capacity for patients with LSS causing NC. In addition, it should increase self-efficacy and decrease anxiety. This will also provide patients with a conservative treatment option that takes into consideration the day-to-day fluctuation in symptom severity. To our knowledge, this is the first LSS-specific rehabilitation program providing standardized education and graded exercises aimed at improving walking capacity regardless of patients’ current healthcare trajectories.

This study protocol also presents innovative components. Indeed, by adding inertial wearable sensors during the SPWT walking evaluation, this study will provide original and accurate information about the gait pattern characteristics of patients with LSS causing NC. By monitoring the gait biomechanical parameters throughout the study, we will also be able to determine which ones are modified by exercises and how they are modified. This could eventually help researchers and clinicians better understand the patient’s biomechanical adaptations over time as well as the impact of specific exercises on walking capacity or gait pattern characteristics. Findings from this study may eventually lead to improvement in the evaluation and management of patients with LSS causing NC.

## Trial status

Version: 1 (July 07, 2022)

Recruitment (anticipate): from January 9, 2023, to December 31, 2024

Study setting:

List of study sites:

1. Chiropratic academic teaching clinic: Université du Québec à Trois-Rivières, Trois-Rivières, Québec, Canada
2. Regional hospital neurosurgery outpatient clinic: Centre intégré universitaire de santé et de services sociaux de la Mauricie-et-du-Centre-du-Québec, Trois-Rivières, Québec, Canada

## Supplementary Information

**Additional file 1.**

## Data Availability

Original consent form, data, and materials are available upon reasonable request to the authors.
